# Modulation of fenestrated vasculature in the median eminence and area postrema in response to neurotoxin exposure and its impairment in aging

**DOI:** 10.3389/fnagi.2025.1634283

**Published:** 2025-08-19

**Authors:** Viana Q. Pham, Melike Tutunculer, Halah Al-Dulaimi, Daniel Ardjmand, William Fleischmann, Tomas P. Bachor, Allison W. Xu

**Affiliations:** Diabetes Center and Department of Anatomy, University of California, San Francisco, San Francisco, CA, United States

**Keywords:** fenestrated capillaries, circumventricular organs, median eminence, area postrema, aging, tanycytes, PTN, VEGF

## Abstract

Effective communication between the brain and peripheral tissues is crucial for homeostasis and health, and its impairment is a defining feature of aging. Circumventricular organs, characterized by the presence of fenestrated capillaries and absence of a blood–brain barrier (BBB), play a crucial role in controlling substance exchange between the brain and the blood. To date, adaptive changes in fenestrated vasculature in response to environmental insults remain poorly understood. In this study, we show that fenestrated capillaries in the median eminence (ME) and area postrema (AP)—two distinct circumventricular organs critical for metabolic control—undergo differential remodeling when exposed to circulating monosodium glutamate (MSG), a BBB-impermeable neurotoxin. Upon MSG exposure, fenestrated capillaries and vascular permeability were decreased in the ME but increased in the AP, and these changes were closely associated with the expression of angiogenic factors pleiotrophin (*Ptn*) and vascular endothelial growth factor A (*Vegfa*). In both ME and AP, adult tanycytes expressed high levels of *Ptn* and have processes in close contact with fenestrated capillaries. Significantly, the adaptive regulation of *Ptn* expression and the ability to modulate fenestrated capillaries and vascular permeability were abolished in both ME and AP of aged animals. Together, our findings suggest that tanycytic expressions of the angiogenic factor PTN, in conjunction with VEGF, are differentially regulated in distinct circumventricular organs upon exposure to neurotoxins, leading to region-specific remodeling of fenestrated endothelium. Our study further demonstrates that the loss of plasticity in fenestrated vasculature may be a hallmark feature of brain aging.

## Introduction

The brain is protected by the blood–brain-barrier (BBB), a barrier along the vasculature consisting of inter-endothelial tight junctions, astrocytic end-feet, pericytes and cellular basement membranes (Abbott et al., 2010). The BBB plays a vital role in regulating the exchange of substances between the brain and the blood while blocking toxins, pathogens, inflammatory cells and antibodies from entering the brain. In contrast, certain brain regions, known as the circumventricular organs, lack a BBB and instead contain fenestrated capillaries, allowing the free passage of circulating molecules up to a certain size through the fenestrated endothelium (Benarroch, 2011; Kaur and Ling, 2017). Located in the mediobasal hypothalamus at the floor of the 3rd ventricle, the median eminence (ME) is classified as a secretory circumventricular organ. The ME extends dorsolaterally into the hypothalamic arcuate nucleus (ARC) and connects ventrally to the infundibular stalk. Fenestrated capillaries are situated at the outer edge of the ME and form distinct microvessel loops extending into the ARC and ME parenchyma. Hypothalamic neuroendocrine neurons project to the ME and release neuroendocrine hormones into the hypothalamic-hypophyseal portal system to regulate growth, reproduction, and metabolism. On the other hand, the area postrema (AP), located in the dorsal vagal complex of the caudal brainstem, is a sensory circumventricular organ. Neurons in the AP detect circulating substances such as toxins, and subsequently act as a chemoreceptor trigger zone for emesis (Han and de Araujo, 2021). In addition, the AP contains neurons that are important for satiety control. Thus, the ME and AP are circumventricular organs with distinct anatomy and metabolic functions (Morita and Miyata, 2012).

Endothelial cells in the BBB of a healthy adult brain have very low turnover rate, ensuring stability and functional integrity of the BBB (Rosenstein and Krum, 1994). In stark contrast, fenestrated capillaries in circumventricular organs of adult animals are continuously remodeled (Morita et al., 2013; Miyata, 2015). Prior studies have shown that vascular endothelial growth factor (VEGF) is involved in the angiogenesis of fenestrated endothelial cells (Morita et al., 2013; Langlet et al., 2013). In the ME, fasting or maternal obesity triggers the elongation and migration of fenestrated microvessel loops into the ARC parenchyma, thereby enhancing vascular permeability (Langlet et al., 2013; Kim et al., 2016). These findings suggest that remodeling of fenestrated blood vessels is a regulated process, dysregulation of which could impair information exchange between the brain and the periphery. Much less is known about how fenestrated capillaries are regulated in the AP.

Aging is a major risk factor for BBB dysfunction, contributing to increased permeability, impaired transport mechanisms, and neuroinflammation (Banks et al., 2021). As the BBB ages, structural and functional changes occur, compromising the regulated exchange of molecules between the brain and the circulation. Such dysfunction is associated with an increased risk of neurodegenerative diseases, including Alzheimer’s and Parkinson’s disease, as well as cognitive decline and vascular dementia (Banks et al., 2021; Erdo et al., 2017; Mooradian, 1988; Shah and Mooradian, 1997). In contrast to the BBB, little is known about the impact of aging on fenestrated vasculature in the circumventricular organs. In this study, we show that fenestrated capillaries in the ME and AP undergo distinct remodeling in response to BBB-impermeable circulating neurotoxins, which damage neurons in the circumventricular organs. Furthermore, tanycytes in the ME and AP express high levels of the angiogenic factor pleiotrophin (PTN), which may play a regulatory role in fenestrated capillary remodeling. Importantly, aged mice fail to exhibit adaptive changes that are observed in young adults, suggesting that the loss of fenestrated vascular plasticity is a hallmark of aging.

## Materials and methods

### Animals

Young (2–4 months) and old (21–25 months) male wild type C57Bl/6 mice were obtained from the NIA colonies at The Jackson Laboratory. The mice were group-housed (12 h light/dark cycle) in a pathogen free environment. Mice were fed standard mouse chow (5,053 - PicoLab® Rodent Diet 20; 20% protein, 4.5% fat). All animal care and experimental protocols were approved by the University of California, San Francisco Institutional Animal Care and Use Committee.

### Monosodium glutamate administration

Mice received one subcutaneous injection of monosodium glutamate (MSG) (3.5 g/kg) or isomolar sodium chloride solution as vehicle (0.02 M/kg or 1.2 g/kg). All solutions were prepared on the day of injection and filter sterilized. One day after injection, mice were perfused and the brains processed for histology.

### Evans blue administration and permeability measurement

Evans blue dye (1% w/v in sterile saline) was prepared on the day of injection and filtered sterilized. Mice were briefly anesthetized using an isoflurane chamber and then quickly administered 50 μL of Evans blue solution retro-orbitally. Perfusion with 4% (w/v) paraformaldehyde (PFA) occurred 20 min later. Direct fluorescence emitted by Evans blue was captured following frozen cryosectioning using an Olympus BX51WI microscope equipped with a QImaging Retiga 2000R digital camera. To measure EB permeability, EB permeable area instead of fluorescence intensity was measured since EB fluorescence intensity includes signals within the blood vessel lumens, intracellular as well as extracellular space, which does not reflect the permeable area. Measured values reflect the area of the ARC or AP with Evans blue dye.

### Immunohistochemistry

Mice were anesthetized with an intraperitoneal injection of Avertin (250 mg/kg; Sigma Aldrich #T48402) followed by perfusion with 4% (w/v) PFA. Brains were post-fixed in 4% PFA, immersed in 30% sucrose overnight, and coronally sectioned at 20 μm on a cryostat. Immunofluorescent staining was performed using the following antibodies: rabbit anti-Vimentin (1:1000, Abcam #ab92547), rat anti-PVLAP (1:200, DHSB #MECA-32-S), rabbit anti-Collagen IV (1:1000, Abcam #ab6586), goat anti-Collagen IV (1:500, Novus Biologicals #NBP1-26549), and mouse anti-GFAP (1:1000, Cell Signaling Technology #3670). Sections underwent heat-mediated antigen retrieval in a 10 mM citrate solution. Following incubation with a blocking solution for 1 h, primary antibodies were added to sections and left overnight at 4 °C. Secondary antibodies were applied for 2 h in the dark at room temperature. Washes with PBS-T (0.1% Triton-X) occurred between each solution.

### RNAscope® *in situ* hybridization

To detect *Vegfa*, *Ptn*, *Vimentin*, *Plvap* mRNA, RNAscope® fluorescent assay (Advanced Cell Diagnostics Inc., Hayward, CA) was employed. The Multiplex Fluorescent Reagent Kit v2 (ACD, Cat no. 323100) was used with the Opal 520 (Akoya Biosciences, Cat no. FP1487001KT, 1:1500), 620 (Akoya Biosciences, Cat no. FP1495001KT, 1:1500), and 690 (Akoya Biosciences, Cat no. FP1497001KT, 1:1500) Reagent Packs. The following probes from ACD were used Mm-Ptn-C3 (Cat no. 486381-C3), Mm-Vegfa-ver2 (Cat no. 412261), Mm-Vim-C3 (Cat no. 457961-C3), and Mm-Plvap-C2 (Cat no. 440221-C2). Sections (20 μm) were imaged on the Leica SP8 inverted laser scanning confocal.

### Data quantification

Data analysis was performed using ImageJ/FIJI. The bregma of brain sections was defined using the Allen Brain Atlas and “The Mouse Brain- In Stereotaxic Coordinates” by Franklin and Paxinos. Images were blinded prior to quantification using *A Better Finder Rename 11* software, which randomized the file order and renamed the files with numeric identifiers.

To obtain tanycyte cell counts, DAPI-positive nuclei along the 3rd ventricular wall were quantified in 63x single-plane images of the mediobasal hypothalamus. To quantify microvessel loops in the hypothalamus, PLVAP-expressing vessels extending from the outer wall of the ME were visualized from Bregma −1.7 to −2.1 to obtain the average count per section. To measure glial and microvessel loop interactions, stained sections were imaged on the Leica SP8 inverted laser scanning confocal microscope. Using binarized single confocal plane images, the area of overlapping pixels between channels for glial cells and blood vessels was defined as an “interaction.” Data were expressed as a percentage of capillary surface coverage, based on Collagen IV area. Interactions were measured along 200 μm of the ME capillary bed for each image. To measure fenestrated capillaries in the AP, a binary threshold was performed on fluorescent images to segment signal-positive regions. Total fluorescent signal area was then quantified as a readout for capillary abundance. Vessels in the AP were visualized from Bregma −7.56 to −7.64.

To quantify mRNA expression in hypothalamic tanycytes, which are densely juxtaposed, fluorescent intensity was measured as a proxy for transcript abundance. To achieve tanycyte subtype specific expression, the DAPI-labeled nuclei of the 3rd ventricle were divided into subtype regions based on morphology and localization. On the floor of the 3 V, β2-tanycytes were defined by the vertical polarity of their cell bodies and extension of their processes onto the ME capillary bed. The border between the β2- and β1-tanycytes was defined where the orientation of the polarity of the cell bodies changed from vertical to angled. The cells lining the ventrolateral portion of the 3 V were considered β1-tanycytes, who project their curved processes at the ME/ARC border. Dorsal to β1-tanycytes, α2-tanycytes were defined as the cells lining the lateral 3 V adjacent to the ARC and ventromedial hypothalamus. In the AP, where tanycyte-like cells are more dispersed, fluorescent signal was binarized, and the area of positive signal represented mRNA expression levels.

### Statistical analysis

An unpaired two-tailed student’s t-test was employed to compare two independent groups. For comparisons involving two genotypes and multiple treatments or conditions, an ordinary or repeated-measures two-way ANOVA with multiple comparisons was utilized. Normality of the data was analyzed using the Shapiro–Wilk test. In the event that a dataset did not pass the normality test, a nonparametric test was used. All statistical analyses were conducted using GraphPad Prism 10.5.0 software (GraphPad Software, Inc., La Jolla, CA, USA).

## Results

### Exposure to circulating neurotoxins results in a reduction of fenestrated capillary microvessels and vascular permeability in young adult mice but not in aged mice

**Figure 1:**
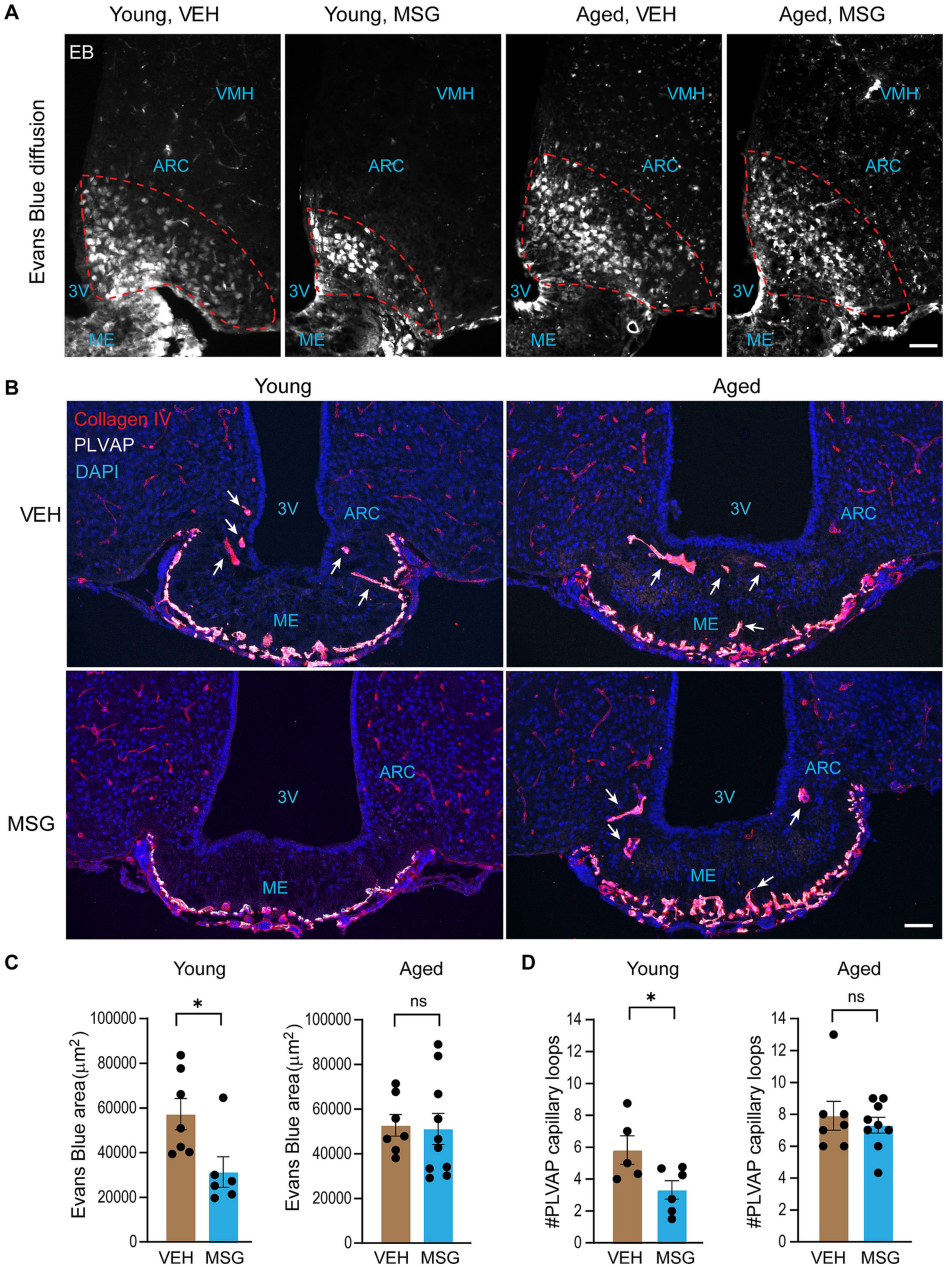
Fenestrated vascular permeability at the mediobasal hypothalamus decreases upon neuronal lesioning in young adults but not aged mice, and is accompanied by a reduction of fenestrated capillary microvessels. Young (2–4 months) and aged (21–25 months) male mice received subcutaneous injections of MSG (3.5 g/kg) or vehicle saline, followed by an injection of Evans blue dye (1% w/v in sterile saline) the next day. **(A)** Representative images of Evans blue penetration into the ARC (outlined areas) of young and old animals. **(B)** Immunostaining for PLVAP to mark fenestrated capillaries and Collagen IV to indicate all blood vessels. White arrows depict fenestrated microvessel loops in the ME and ARC parenchyma. **(C)** Quantification of Evans blue permeable area in the ARC parenchyma (outlined areas). Bregma: −1.7 to −2.1 (2–6 sections/mouse). **(D)** Quantification of the number of PLVAP-positive fenestrated capillary microvessel loops in the ME and ARC parenchyma. Scale bar = 50 μm. Bregma: −1.9 to −2.1 (1–4 sections/mouse). All experiments were conducted using male mice. Data are represented as the mean ± SEM. *p < 0.05, ns, not significant by two-tailed student’s *t*-test or nonparametric test in the event that data did not pass normality test. VEH, vehicle; MSG, monosodium glutamate; ME, median eminence; ARC, arcuate nucleus; 3 V, third ventricle; VMH, ventromedial hypothalamus.

We have previously shown that hypothalamic neurons outside the BBB, but not those that are protected by the BBB, are rapidly ablated by peripheral administration of MSG, a glutamate that does not cross the BBB and ablates neurons by excitotoxicity (Yulyaningsih et al., 2017). We sought to determine if fenestrated vascular permeability in the ME was modulated upon exposure to MSG, and if aging affected such response. To this end, young (2–4 months) and old (21–25 months) male mice were injected subcutaneously with MSG or vehicle (isomolar sodium chloride). One day later, mice were injected with Evans blue, a BBB-impermeable fluorescent dye, right before perfusion. Consistent with what we reported previously (Yulyaningsih et al., 2017; Olofsson et al., 2013), cells in the ME and mediobasal ARC were positive for Evans blue (Figure 1A). Intriguingly, the area of Evans blue permeability in the ARC parenchyma significantly decreased 1 day after MSG treatment in young adult mice but not in aged mice (Figures 1A,C).

Since fenestrated capillaries permit the permeation of Evans blue into the ME and ARC, we visualized fenestrated capillaries by immunostaining of plasmalemma vesicle-associated protein (PLVAP), the protein that makes up the sieve-like diaphragm of the fenestrae in fenestrated endothelial cells and hence specifically marks fenestrated blood vessels (Stan et al., 2012; Denzer et al., 2023). All blood vessels, both fenestrated and non-fenestrated, were visualized by immunostaining with Collagen IV, a component of the basement membrane of blood vessels. PLVAP-positive capillaries were located at the outer edge of the ME with microvessal loops extending into the ME and ARC parenchyma (Figure 1B). Numbers of these PLVAP^+^ fenestrated microvessal loops were reduced 1 day after MSG treatment in young adults but not in aged male mice (Figures 1B,D). Together, these results suggest that the abundance of fenestrated microvessel loops and consequently fenestrated vascular permeability at the ME and ARC decrease upon MSG exposure. Importantly, this regulation is abolished in aged animals, suggesting that aging impairs the ability of fenestrated blood vessels to remodel in response to circulating neurotoxins.

### Tanycytes, but not astrocytes, exhibit a high degree of physical contact with fenestrated capillaries in the median eminence

**Figure 2:**
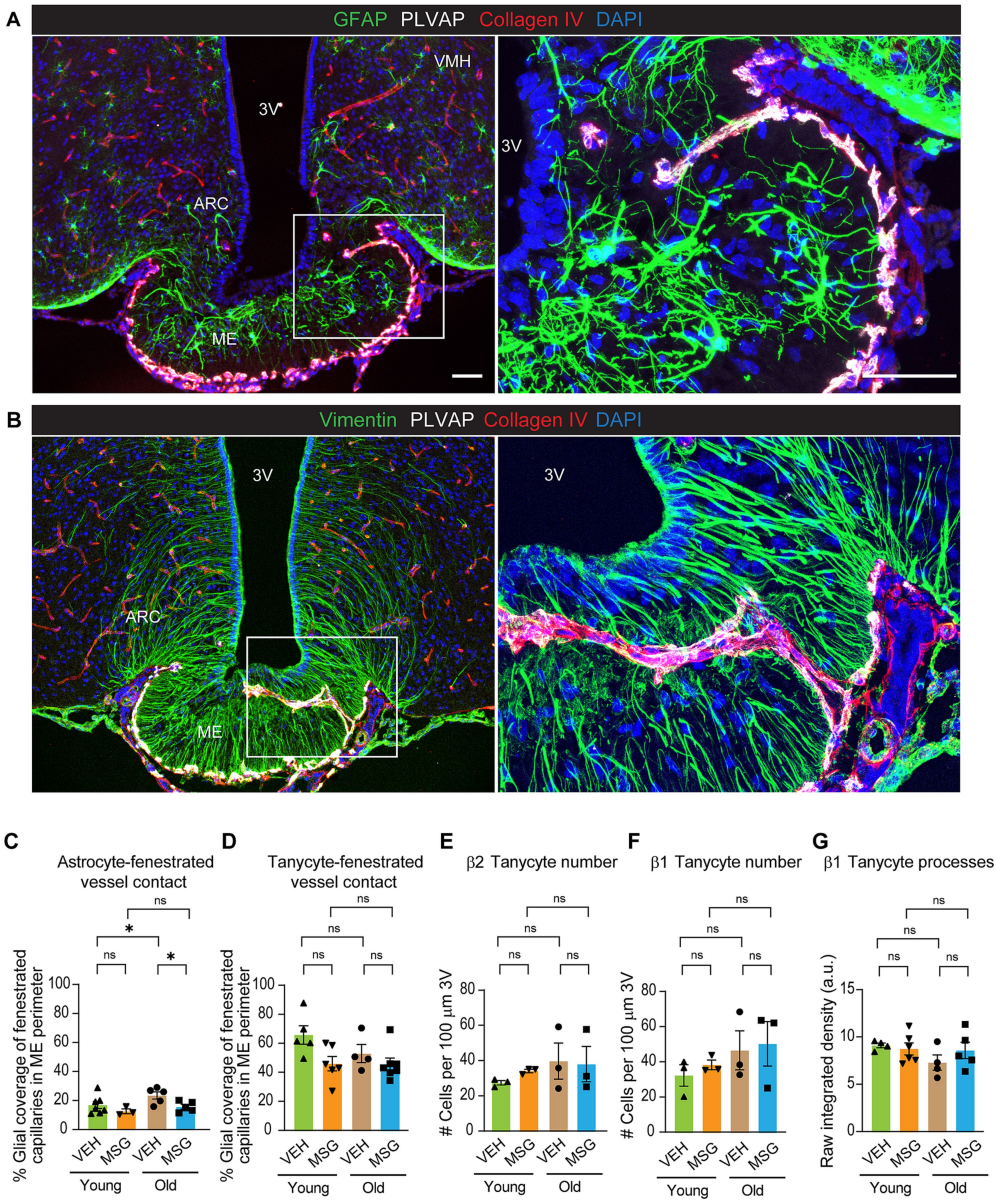
Tanycytes, but not astrocytes, exhibit a high degree of physical contact with fenestrated capillaries in the ME. Young (2–4 months) and aged (21–25 months) mice received subcutaneous injections of MSG (3.5 g/kg) or vehicle saline. Hypothalamic sections were processed 1 day later for immunofluorescent analysis. Basement membranes of all blood vessels were visualized with Collagen IV; fenestrated capillaries were visualized with PLVAP expression; astrocytes were visualized with GFAP, whereas tanycytes were visualized with Vimentin immunoreactivity. **(A)** Representative confocal Z-projection image showing astrocytes, fenestrated and non-fenestrated capillaries. **(B)** Representative confocal Z-projection image showing tanycytes, fenestrated and non-fenestrated capillaries. **(C,D)** Quantification of astrocytic and tanycytic contact with fenestrated capillaries in the ME perimeter. Single-plane confocal images were used for quantification. Bregma: −1.9 to −2.1 (1–2 sections/mouse). **(E,F)** Quantification of beta-tanycyte cell numbers in VEH or MSG treated young and aged mice. **(G)** Abundance of β1 tanycyte processes. Bregma: −1.8 (1 section/mouse). All experiments were conducted using male mice. Scale bars = 50 μm. Bregma: −1.9 to −2.1. Data are represented as the mean ± SEM. *p < 0.05, ***p < 0.001, ns, not significant, by 2-way ANOVA or nonparametric test in the event that data did not pass normality test. VEH, vehicle; MSG, monosodium glutamate; ME, median eminence; ARC, arcuate nucleus; 3 V, third ventricle; VMH, ventromedial hypothalamus.

Glial cells play essential roles in regulating vascular function in the brain. In most brain regions, astrocyte endfeet form a complex network surrounding barrier endothelial cells and this close interaction is vital for the integrity and functionality of the BBB (Abbott et al., 2010). The hypothalamus also contains tanycytes, which are specialized radial glia-like cells that line the wall of the 3^rd^ ventricle. Tanycyte cell bodies are located on the ventricular wall, and each tanycyte extends a single long basal process into the ME or hypothalamic parenchyma (Rodriguez et al., 2005; Langlet et al., 2013). As glial processes may serve as a physical barrier to restrict the diffusion of blood-borne substances from the ME into the ARC parenchyma, we examined the physical interaction between astrocyte or tanycyte endfeet and fenestrated capillaries. Hypothalamic sections from young adult male mice were immunostained with a PLVAP antibody to mark fenestrated capillaries, a Collagen IV antibody to visualize the basement membrane of all blood vessels, a GFAP antibody to label astrocytes, and a Vimentin antibody to reveal tanycytes (Figures 2A,B). Single-plane confocal images were processed and the area of contact between endfeet of astrocytes or tanycytes and fenestrated capillaries in the ME perimeter was quantified (Supplementary Figure 1). Fenestrated capillaries exhibited a strikingly high degree of physical contact with tanycytes, and to a much lesser extent with astrocytes (Figures 2C,D; Supplementary Figure 1). The high degree of physical contact between tanycyte endfeet and fenestrated capillaries suggests that tanycytes may exert significant influence on fenestrated endothelial cells.

### Tanycytic contact with fenestrated capillaries or tanycyte numbers did not change significantly with age or MSG treatment

Astrocytic contact with fenestrated capillaries increased with age, while tanycytic contact did not change. There was also no change in astrocytic contact or tanycytic contact with fenestrated capillaries in young mice in response to MSG treatment (Figures 2C,D; Supplementary Figure 2). Neither MSG treatment or age affected β1, β2 tanycyte numbers (Figures 2E,F; Supplementary Figure 2), or β1 tanycyte processes which may act as a physical barrier between ME and ARC (Figure 2G). Thus, these data do not support the notion that MSG-induced reduction in fenestrated vascular permeability in young mice is attributed to gross structural changes of tanycytes or astrocytes.

### Tanycytes express high levels of angiogenic factors VEGF and PTN, expressions of which are modulated by MSG in young adults but not in aged mice

Since tanycytes show close contact with fenestrated capillaries, they may regulate fenestrated vasculature remodeling through secreted angiogenic factors. We examined a list of tanycyte-enriched genes from published single-cell RNA-sequencing data from the adult hypothalamus (Chen et al., 2017). *Vegfa* was one of such genes (#41 on the list) [Table S4 in Chen et al. (2017)]. Notably, *Ptn* was identified as a top tanycyte-enriched gene (#2 on the list) [Table S4 in Chen et al. (2017)]. PTN, also named heparin affinity regulatory peptide (HARP) in some studies, is a potent angiogenic factor, which stimulates proliferation and migration of endothelial cells during normal development and in cancer (Zhang and Deuel, 1999; Perez-Pinera et al., 2007; Perez-Pinera et al., 2008; Papadimitriou et al., 2022; Papadimitriou et al., 2009; Mikelis and Papadimitriou, 2008; Mikelis et al., 2007). *In vitro* studies show that PTN acts on endothelial cells to stimulate their migration and promote their formation of tube-like structures (Papadimitriou et al., 2001; Souttou et al., 2001). *In vivo*, PTN alone is sufficient to initiate new blood vessel formation that is both normal in appearance and function (Christman et al., 2005). PTN expression is high in the developing brain, peaks at birth but declines as the brain matures (Kadomatsu and Muramatsu, 2004). Despite this, expression and function of PTN in the adult brain are not well characterized.

**Figure 3:**
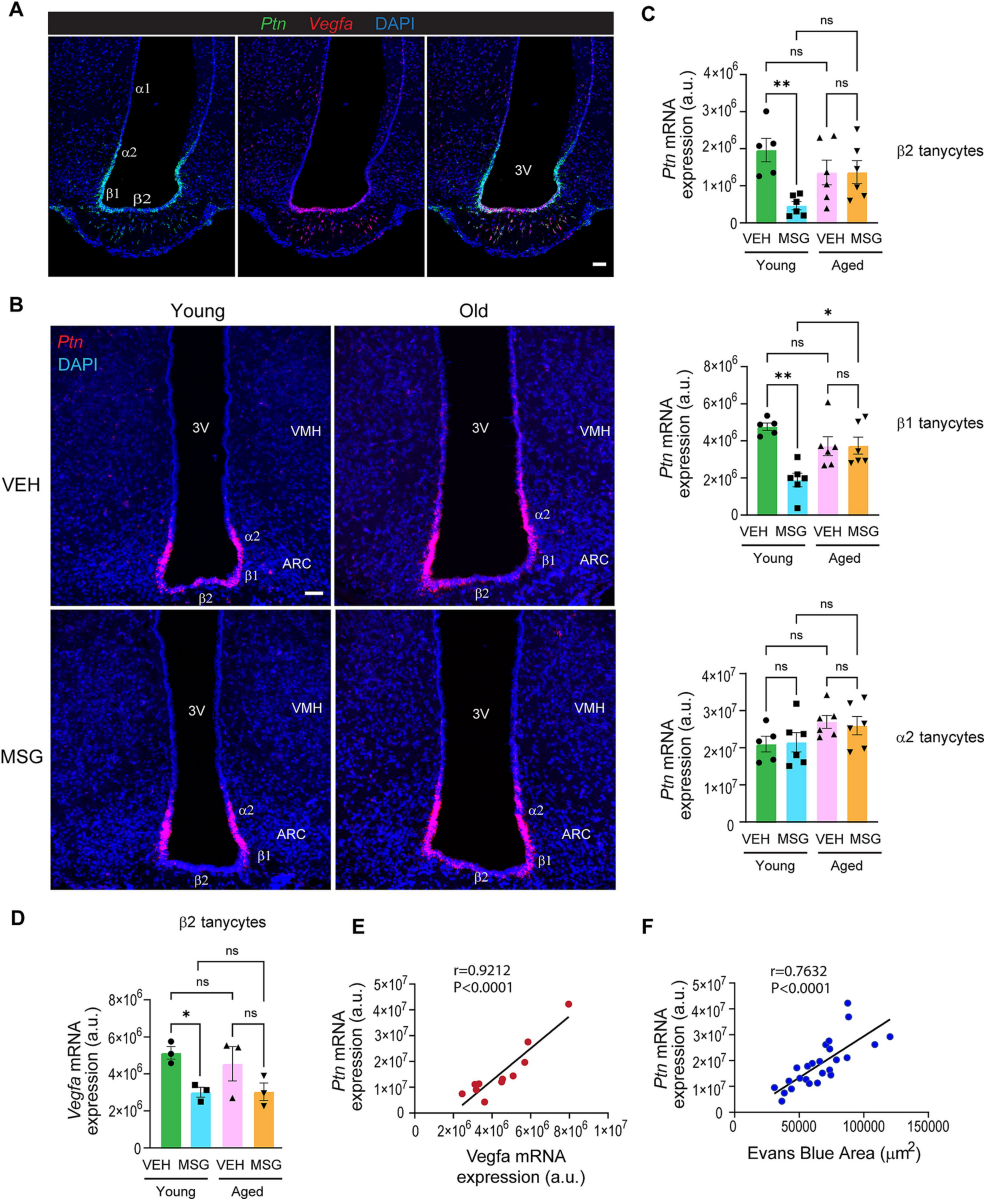
Tanycytic expression of angiogenic factors Ptn and Vegfa undergo remodeling upon exposure to neurotoxin in young adults but not in aged mice. **(A)** *Ptn* and *Vegfa* mRNA expression in adult tanycytes by RNAscope. **(B)** Representative images of the RNAscope analysis for *Ptn* mRNA in young and aged mice treated with VEH or MSG. (**C**) Quantification of *Ptn* expression in tanycyte subtypes in young (2–4 months) and aged (21–25 months) VEH or MSG injected mice by 2-WAY ANOVA or nonparametric test in the event that data did not pass normality test. Bregma: −1.8, −1.9 (1 section/mouse). **(D)** Quantification of *Vegfa* expression in β2 tanycytes by 2-WAY ANOVA. **(E)** Correlation of total *Ptn* and *Vegfa* mRNA expression in all tanycytes using linear regression analysis. **(F)** Correlation of total *Ptn* mRNA expression in all tanycytes with Evans blue diffusion area in the mediobasal arcuate nucleus using linear regression. All experiments were conducted using male mice. Scale bars = 50 μm. Data are represented as the mean ± SEM. *p < 0.05, **p < 0.01, ***p < 0.001, ****p < 0.0001, ns: no significance. VEH, vehicle, MSG, monosodium glutamate; ARC, arcuate nucleus; VMH, ventromedial hypothalamus; ME, median eminence; 3 V, third ventricle. α1, α2, β1, and β2 are subtypes of tanycytes.

We next examined *Ptn* and *Vegfa* mRNA expression in tanycytes of the adult hypothalamus of male mice by RNAscope. Tanycyte cell bodies line the 3^rd^ ventricular wall and are classified into 4 different subtypes based on their locations: α1 tanycytes line the most dorsal wall of the 3^rd^ ventricle, whereas α2 tanycytes occupy the ventral ventricular wall adjacent to the ARC; β1, β2 tanycytes line the lateral evaginations and floor of the 3^rd^ ventricle, respectively. *Ptn* mRNA expression was abundant but restricted to α2, β1 and β2 tanycytes of adult mice, whereas *Vegfa* expression was most highly expressed in β2 tanycytes (Figure 3A). *Ptn* expressions in β1 and β2 tanycytes, cells that directly contact fenestrated capillaries with their endfeet, were reduced in young adult mice after MSG-induced lesioning, and such regulation was abolished in the aged mice (Figure 3C). In contrast, *Ptn* expression in α2 tanycytes, which project into the hypothalamic parenchyma, was not altered by MSG treatment in either young or aged mice (Figure 3C). *Vegfa* expression in β2 tanycytes also followed a similar trend (Figure 3D). Notably, tanycytic *Ptn* expression significantly correlated with *Vegfa* expression (Figure 3E) and with Evans blue permeability in the ME and mediobasal ARC (Figure 3F). Together, these results suggest that expressions of angiogenic factors VEGF and PTN are modulated in response to MSG. As the basal processes of β1 and β2 tanycytes directly contact the fenestrated capillaries, our results suggest that tanycytic release of angiogenic factors VEGF and PTN may regulate the remodeling of fenestrated blood vessels in response to circulating neurotoxins. Our finding also suggests that aging impairs the ability of tanycytes to modulate the expression of angiogenic factors, which may underlie the loss of fenestrated capillary plasticity in aging.

### Fenestrated capillary permeability increases in the area postrema in response to MSG in young adults but not in aged mice

**Figure 4:**
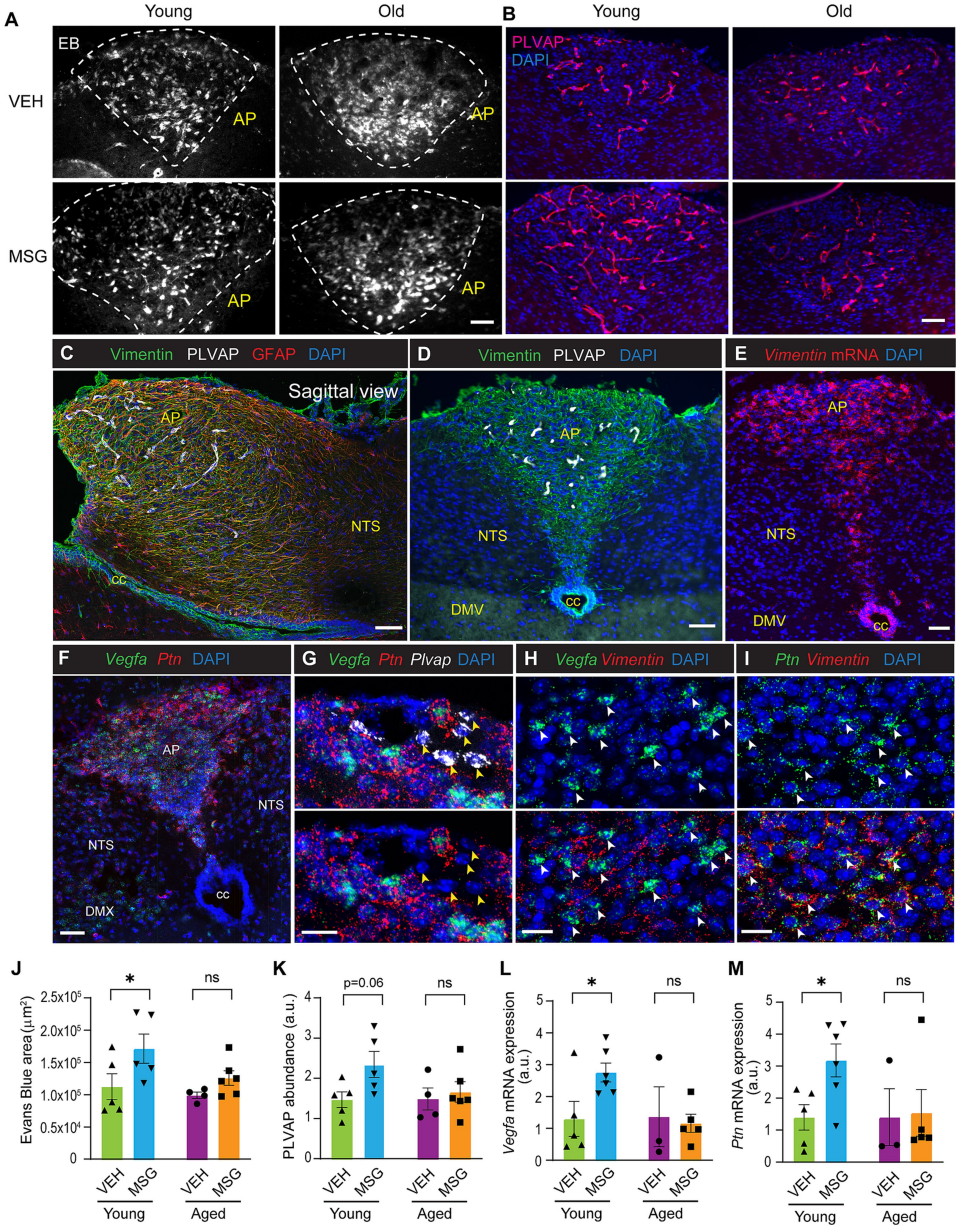
Fenestrated capillary permeability increases in the area postrema in response to MSG in young adults, but not in aged mice. **(A)** Representative images of Evans blue permeability of young and old mice treated with vehicle or MSG. **(B)** PLVAP immuno-positive capillaries of young and old mice treated with vehicle or MSG. **(C)** Glial cells co-expressing markers for tanycyte-like cells and astrocytes in a sagittal view of the AP. **(D)** Immunostaining of Vimentin and PLVAP. **(E)** RNAscope for *Vimentin* mRNA. **(F)** RNAscope for *Vegfa* and *Ptn* mRNA localized expression in the AP. **(G)** RNAscope for *Vegfa, Ptn* and *Plvap* mRNA in the AP. Yellow arrowheads indicate *Plvap*-positive endothelial cells. **(H)** RNAscope for *Vegfa and Vimentin* mRNA in the AP. White arrowheads indicate *Vegfa-*expressing cells. **(I)** RNAscope for *Ptn and Vimentin* mRNA in the AP. White arrowheads indicate *Ptn-*expressing cells. **(J,K)** Quantification of Evans blue permeability and PLVAP immuno-positive capillaries in the AP of young and old mice treated with vehicle or MSG. Images were shown in Panels **A,B**. **(L,M)** mRNA expression of *Vegfa* and *Ptn* in the AP of young and old mice treated with vehicle or MSG. Bregma: −7.48 to −7.56 (1–3 sections/mouse). All experiments were conducted using male mice. Scale bars: 50 μm for Panel A-F, 20 μm for Panels **G–I**. Panels **J–M**: Data are represented as the mean ± SEM. *p < 0.05, ns: no significance by 2-WAY ANOVA or nonparametric test in the event that data did not pass normality test. VEH, vehicle; MSG, monosodium glutamate; AP, area postrema; NTS, nucleus of tractus solitarius; cc, central canal; DMX, dorsal motor nucleus of the vagus.

To evaluate if MSG-induced remodeling of fenestrated vasculature is unique to the ME, we examined the AP, another circumventricular organ in the brainstem in male mice. Surprisingly, the Evans blue permeable region in the AP of young adult mice was not reduced but rather increased in response to MSG treatment and again this modulation was significantly diminished in the aged mice (Figures 4A,J). Consistently, the abundance of PLVAP-positive fenestrated capillaries in the AP were increased upon MSG treatment in young adults but not in aged mice (Figures 4B,K). Vimentin-positive tanycyte-like cells were present in the AP (Figures 4C–E), but unlike the *β* tanycytes at the ME which do not express GFAP, tanycyte-like cells in the AP co-expressed Vimentin and GFAP (Figure 4C). Notably, mRNA expression of *Vegfa* and *Ptn* were highly enriched in the AP (Figure 4F). *Vegfa* and *Ptn* were not expressed by *Plvap^+^* endothelial cells (Figure 4G). Only about 40% of *Vegfa*-expressing cells in the AP expressed *Vimentin* (Figure 4H). However, near 80% *Ptn*-expressing cells co-expressed *Vimentin*, suggesting that PTN is mainly produced by tanycyte-like cells in the AP (Figure 4I). Notably, both *Vegfa* and *Ptn* expression were increased upon MSG treatment, and this regulation was abolished in aged mice (Figures 4L,M). Together, these results suggest that fenestrated capillaries in the AP undergo distinct remodeling compared with those in the ME upon exposure to circulating neurotoxins and that this regulation is impaired in aging. The concordant regulation of *Vegfa* and *Ptn* expression suggests that modulation of angiogenic factors in the AP may play a key role in the remodeling of fenestrated vasculature in this region and that this regulation is lost in aged animals.

## Discussion

Proper communication between the brain and the periphery is essential to maintain homeostasis and health. Circumventricular organs, characterized by the presence of fenestrated capillaries and absence of a BBB, play crucial roles in regulating the exchange of substances between the brain and the blood. In this study, we show that fenestrated vasculature in the ME and AP, two different circumventricular organs that are important for metabolic control, undergo distinct remodeling in response to the circulating toxin MSG, and that this process could be controlled by tanycytic release of angiogenic factors. We further show that a loss of plasticity in fenestrated vasculature in both ME and AP is a defining feature of aging.

One intriguing finding of this study is that fenestrated vascular permeability in the ME and AP undergoes changes in opposite fashion in response to MSG. The ME is classified as a secretory circumventricular organ, where hypothalamic axonal terminals project and release neuroendocrine hormones into the hypophyseal portal vessels. Hypothalamic neuroendocrine hormones such as gonadotropin-releasing hormone and growth hormone-releasing hormone regulate growth and reproduction. Thus, a reduction in fenestrated vascular permeability in the ME in response to environmental insults would limit the release of neuroendocrine hormones into the portal circulation, thereby suppressing growth and reproduction during a period of brain repair. In contrast to the ME, the AP is classified as a sensory circumventricular organ. In species capable of emesis, neurons in the AP detect circulating toxins and other harmful substances in the body, triggering nausea and vomiting to expel them (Han and de Araujo, 2021). Activation of AP neurons by a circulating toxin also suppresses appetite, thus limiting the ingestion of potentially harmful substances (Zhang et al., 2021). Thus, an increase in fenestrated vascular permeability in the AP may allow maximal exposure of AP neurons to the blood thereby enhancing their sensory function.

Unlike endothelial cells in the BBB, fenestrated endothelial cells within circumventricular organs undergo constant remodeling in the adult brain (Rosenstein and Krum, 1994; Morita et al., 2013; Miyata, 2015). Our study suggests that tanycyte-derived angiogenic factor PTN may play a regulatory role in modulating fenestrated capillaries in the ME and AP, and that PTN may act in concert with VEGF. Tanycytes are specialized glial cells and are uniquely localized within circumventricular organs. Their close physical contact with fenestrated capillaries suggests that tanycytes could play important roles in modulating fenestrated endothelial cells. It has been shown that tanycytic secretion of VEGF in the ME promotes elongation and migration of fenestrated microvessel loops into the ARC parenchyma in response to fasting (Langlet et al., 2013). Our study shows that tanycytes also express abundant PTN, a growth factor that has potent angiogenic activity (Zhang and Deuel, 1999; Perez-Pinera et al., 2007; Perez-Pinera et al., 2008; Papadimitriou et al., 2022; Papadimitriou et al., 2009; Mikelis and Papadimitriou, 2008; Mikelis et al., 2007; Papadimitriou et al., 2001; Souttou et al., 2001; Christman et al., 2005). PTN expression is high in the developing brain, peaks at birth but then declines as the brain matures (Kadomatsu and Muramatsu, 2004). However, we show that PTN expression remains high in tanycytes of the adult brain, and that its expression is modulated in response to MSG in both the ME and AP. Future experiments will determine if tanycytic PTN may function synergistically or differently with VEGF to regulate different aspects of fenestrated endothelial cell remodeling such as endothelial cell proliferation, survival, and migration.

Our results show that tanycytes exhibit greater physical contact with fenestrated capillaries compared with astrocytes (Figures 2A–D), In addition, the highest levels of *Ptn* and *Vegfa* expression were in cells on the wall of the ventral 3^rd^ ventricle, where tanycytes are located (Figure 3A). These data suggest that tanycytes could be an important modulator of fenestrated endothelial cells through the release of angiogenic factors. However, it should be emphasized that VEGF and PTN can be expressed by non-tanycyte cell types in the brain, which may influence fenestrated endothelial cells. Future experiments are required to determine if tanycytes are indispensable for this regulation, and to assess the relative contribution of different cell types.

Currently, it is not known how tanycytes sense the effects of MSG. Glutamate is an excitatory neurotransmitter and exerts excitotoxic effects at high concentrations, and MSG does not cross the BBB. Injecting MSG into neonatal monkeys or rodents causes rapid hypothalamic neuronal degeneration within hours (Olney and Sharpe, 1969; Olney, 1969; Burde et al., 1971). Thus, it is possible that tanycytes may sense signals coming from degenerating neurons. Alternatively, β2 tanycytes express glutamate receptors (Farkas et al., 2020), suggesting that tanycytes may be able to directly respond to MSG. Thus, tanycytes might sense MSG directly or indirectly, leading to altered expression of angiogenic factors, subsequently impacting fenestrated endothelial cells.

It is well recognized that aging is associated with histological changes in components of the brain capillaries, and that BBB permeability increases in neurological diseases such as Alzheimer’s and Parkinson’s disease (Erdo et al., 2017; Mooradian, 1988; Shah and Mooradian, 1997). The consequence of aging on fenestrated vasculature in the brain is not well known. One key finding of this study is the demonstration that the plasticity of fenestrated vascular remodeling is lost in aging. This loss of fenestrated capillary plasticity abolishes the ability of aged animals to mount an adaptive response following exposure to circulating toxins or other physiological insults, which would compromise the dynamic communication between the brain and blood in the circumventricular organs. Our results suggest that tanycyte number and processes do not change significantly with age; rather, tanycytes in aged mice have lost the ability to regulate expression of angiogenic factors in response to environmental insult. As the ME and the AP are key brain areas for metabolic control, dysregulation of tanycytic function could be an important mechanism contributing to brain aging and the diminished ability to maintain homeostasis in aged animals.

## Data Availability

The original contributions presented in the study are included in the article/Supplementary material, further inquiries can be directed to the corresponding author/s.
