# Supplementary figures and images for: Modulation of fenestrated vasculature in the median eminence and area postrema in response to neurotoxin exposure and its impairment in aging
Source: Front Aging Neurosci. 2025 Aug 19;17:1634283. doi: 10.3389/fnagi.2025.1634283 (PMC12401994; doi:10.3389/fnagi.2025.1634283)

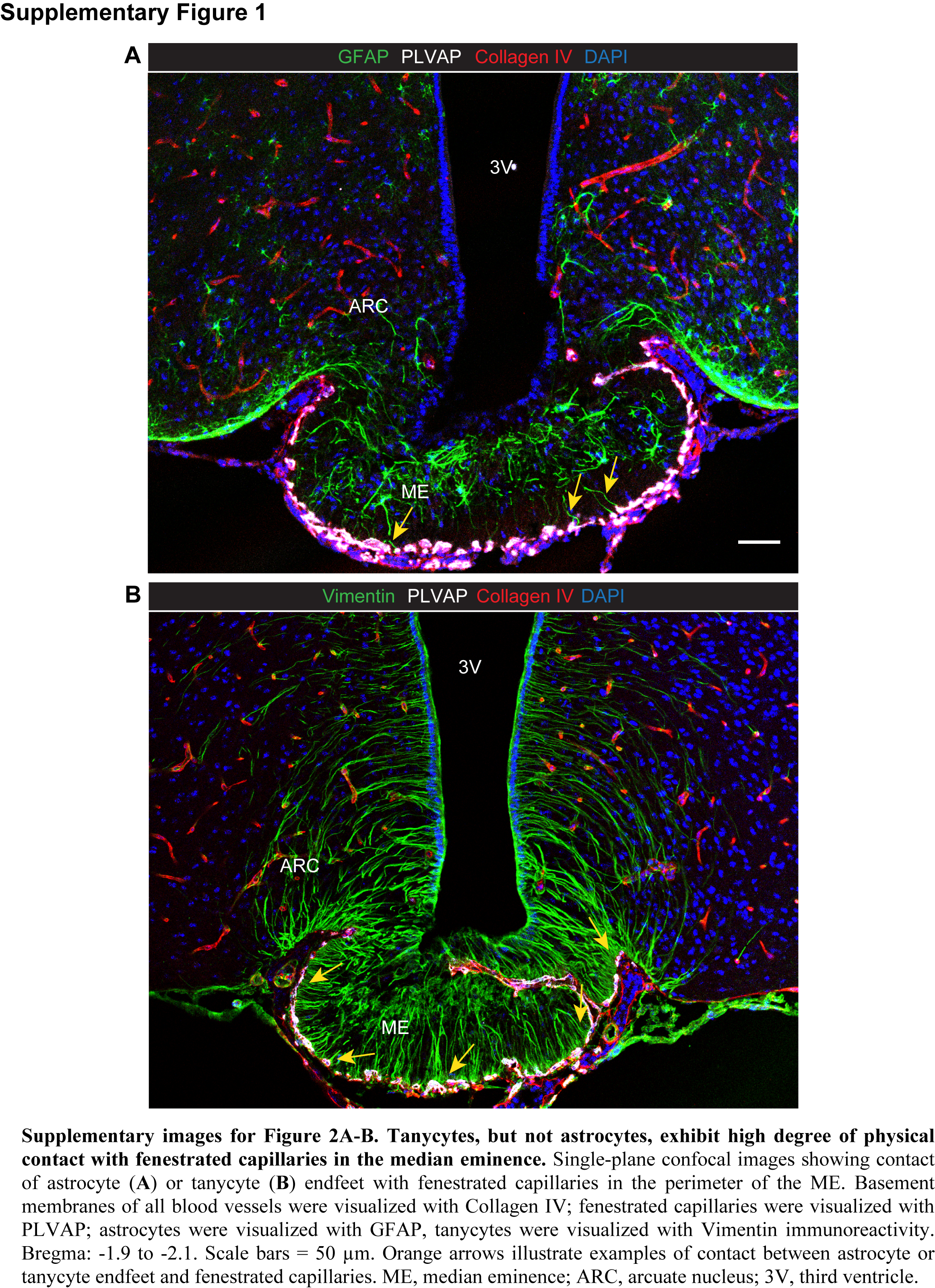

Supplement: Supplementary file 1 [file Image_1.tif]

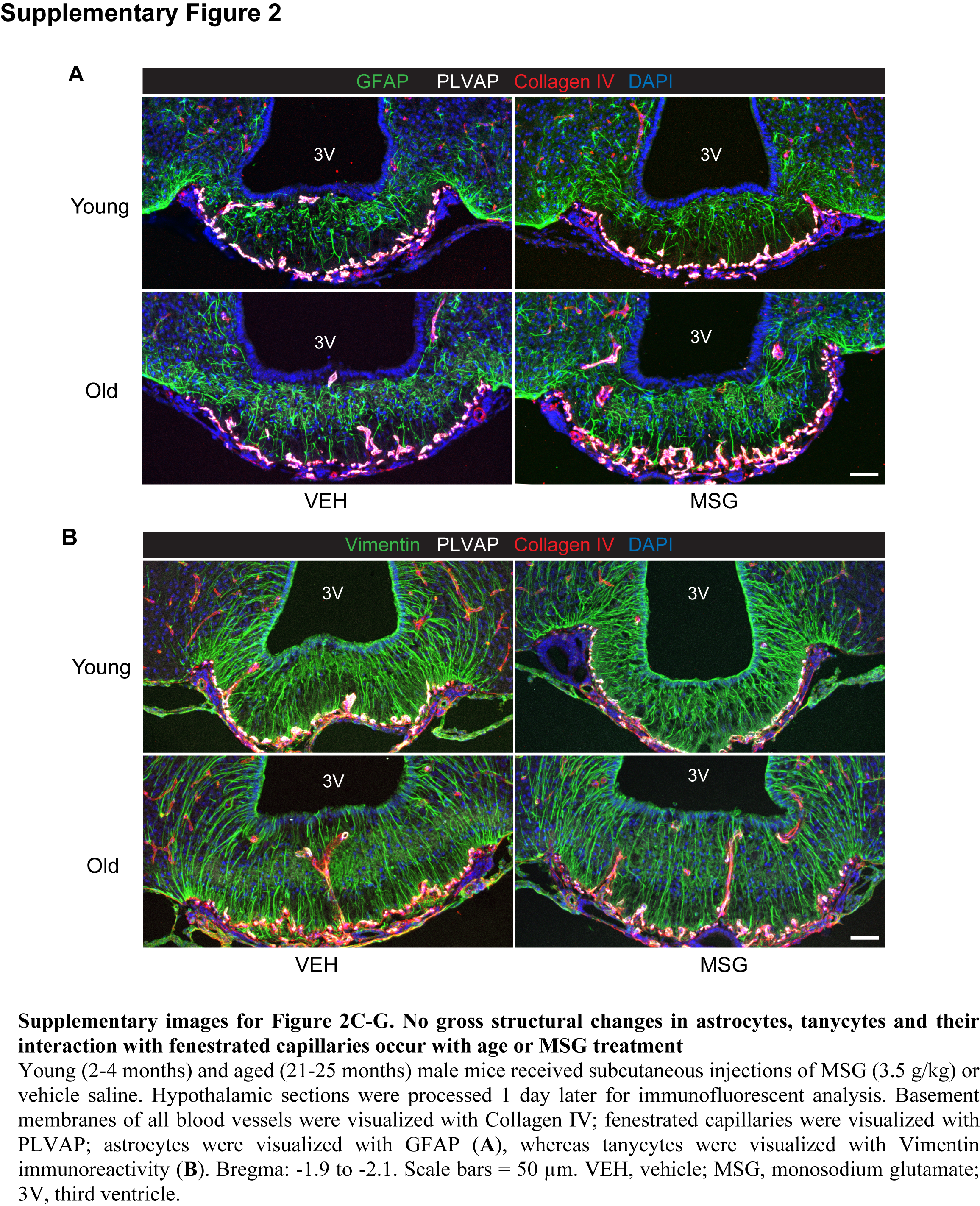

Supplement: Supplementary file 2 [file Image_2.tif]
